# Effects of Dinoflagellate Toxins Okadaic Acid and Dinophysistoxin-1 and -2 on the Microcrustacean *Artemia franciscana*

**DOI:** 10.3390/toxins17020080

**Published:** 2025-02-10

**Authors:** Federica Cavion, Silvio Sosa, Jane Kilcoyne, Alessandra D’Arelli, Cristina Ponti, Michela Carlin, Aurelia Tubaro, Marco Pelin

**Affiliations:** 1 Department of Life Sciences, University of Trieste, 34127 Trieste, Italy; federica.cavion@hotmail.it (F.C.); cponti@units.it (C.P.); michela.carlin@units.it (M.C.); tubaro@units.it (A.T.); mpelin@units.it (M.P.); 2 Marine Institute, Rinville, Oranmore, H91 R673 County Galway, Ireland; jane.kilcoyne@marine.ie

**Keywords:** diarrhetic shellfish poisoning toxins, okadaic acid, dinophysistoxins, *Artemia franciscana*, ecotoxicology, oxidative stress, antioxidant enzyme activity

## Abstract

Harmful algal blooms are an expanding phenomenon negatively impacting human health, socio-economic welfare, and ecosystems. Such events increase the risk of marine organisms’ exposure to algal toxins with consequent ecological effects. In this frame, the objective of this study was to investigate the ecotoxicological potential of three globally distributed dinoflagellate toxins (okadaic acid, OA; dinophysistoxin-1, DTX-1; dinophysistoxin-2, DTX-2) using *Artemia franciscana* as a model organism of marine zooplankton. Each toxin (0.1–100 nM) was evaluated for its toxic effects in terms of cyst hatching, mortality of nauplii Instar I and adults, and biochemical responses related to oxidative stress. At the highest concentration (100 nM), these toxins significantly increased adults’ mortality starting from 24 h (DTX-1), 48 h (OA), or 72 h (DTX-2) exposures, DTX-1 being the most potent one, followed by OA and DTX-2. The quantitation of oxidative stress biomarkers in adults, i.e., reactive oxygen species (ROS) production and activity of three endogenous antioxidant defense enzymes (glutathione S-transferase, superoxide dismutase, and catalase) showed that only DTX-2 significantly increased ROS production, whereas each toxin affected the antioxidant enzymes with a different activity profile. In general, the results indicate a negative impact of these toxins towards *A. franciscana* with potential consequences on the marine ecosystem.

## 1. Introduction

Harmful algal blooms (HABs), occurring worldwide in marine, estuarine, and freshwater environments, are known for their significant negative impact on human health, socio-economic welfare, and aquatic ecosystems. Numerous marine harmful algal species can cause direct adverse effects on aquatic organisms or their toxins can be accumulated by different species and transferred along the food web, with negative effects at higher trophic levels [1]. Algal toxins can impact human health through contaminated seafood consumption or other exposure routes, such as skin contact with seawater and/or the inhalation of marine aerosols during recreational or occupational coastal activities. These events can significantly impact socio-economic systems, including public health costs, aquaculture and seafood industries, and the tourism sector [2].

More than 140 potentially harmful algal species or algal-like bacteria are reported in the IOC-UNESCO taxonomic reference list [3], the main toxin producers being Cyanobacteria, dinoflagellates, Diatoms, Raphidophytes, Pelagophytes, and Haptophytes [4]. The major marine phycotoxins contaminating edible shellfish or fish were originally classified according to their adverse effects in humans, i.e., toxins causing Paralytic Shellfish Poisoning (PSP), Neurotoxic Shellfish Poisoning (NSP), Amnesic Shellfish Poisoning (ASP), Azaspiracid Shellfish Poisoning (AZP), Diarrhetic Shellfish Poisoning (DSP), and Ciguatera Fish Poisoning (CFP) [5]. Among them, the globally distributed DSP toxins, produced by dinoflagellates of the genera *Dinophysis* and *Prorocentrum,* are involved in the majority of algal-related harmful events affecting humans [6,7].

The main DSP toxins are okadaic acid (OA), dinophysistoxin (DTX)-1, DTX-2, and DTX-3 (a heterogeneous group of 7-*O*-acyl esters of OA, DTX-1, and/or DTX-2, formed by the metabolic transformation of the latter in shellfish) [8,9,10]. The main representative DSP toxin is OA, a long-chain heat-stable lipophilic polyether, characterized by a carboxyl group, that in aqueous solutions folds on itself to form a pseudo-ring structure with a hydrophobic tail. DTX-1 and DTX-2 differ from OA only in the number of the methyl groups in C31 and C35, and in the stereochemistry in C35, DTX-1 being 35(*R*)-methyl OA, and DTX-2 being 35(*S*)-methyl-31-desmethyl OA. Given its lipophilic nature, OA can easily cross the phospholipid bilayer of cell membranes [11,12], suggesting that DSP toxins can reach their intracellular molecular targets, i.e., serine/threonine protein phosphatases. OA, DTX-1, and DTX-2 are potent inhibitors of protein phosphatase 2A (PP2A) as well as protein phosphatase 5 (PP5) and 1 (PP1), with the following order of potency: PP2A > PP5 > PP1 [13]. Their inhibitory potency on a recombinant and a wild-type PP2A allowed the determination of DTX-1 and DTX-2 inhibition equivalency factors in comparison to OA, equal to 1.1 and 0.9 (DTX-1) or 0.4 and 0.6 (DTX-2) for recombinant and wild-type PP2A, respectively [14]. Protein phosphatases are essential for the control of many cellular processes, such as signal transduction, cell division and differentiation, muscle cell contraction, neuronal activity, and metabolism [15]. Studies on OA showed that its binding to cell protein phosphatases leads to a rapid increase in the phosphorylation level of cell proteins, including those controlling the secretion of sodium ions. This effect seems to be involved in diarrhea development and other gastrointestinal symptoms characterizing DSP [16], even though other secretory mechanisms have been suggested [17,18].

While numerous studies investigated the effects of DSP toxins on mammals, only a few studies evaluated their effects on other animals, in particular of the marine ecosystem. Most of them have been carried out on edible bivalve mollusks due to their high commercial value and their surprising resistance to these toxins. For example, some studies evaluated the genotoxicity of OA on clams (*Ruditapes decussatus*). In vitro studies on clam hemocytes showed a rapid genotoxic effect of OA. In contrast, exposure of the whole organism to OA caused genetic material damage only in the gill tissue, even at low OA concentrations [19]. The induction of micronuclei as a genotoxic effect has also been recorded in bivalves, due to the loss of chromosomal fragments or entire chromosomes [20]. However, a decreased frequency of micronuclei has also been observed in some bivalves exposed to high OA concentrations, probably due to the activation of cell repair mechanisms [21].

Additionally, the gills of seabream (*Sparus aurata*) have been damaged by short-term exposure to OA, as shown by hypertrophy, fusion in secondary lamellae, and necrosis [22]. Other studies used embryos of zebrafish (*Danio rerio*) or medaka fish (*Oryzias* species) as experimental models to assess OA effects on embryo development. Anatomopathological analyses on the surviving medaka embryos indicate that exposure to OA can induce diverse alterations, such as a significant increase in the liver and digestive tract areas in *Oryzias latipes* embryos [23], or spinal curvature, dysplasia, and tail curvature in *Oryzias melastigma* embryos [24]. Furthermore, OA and DTX-1 induced pathological alterations in zebrafish larvae, such as pericardial edema, cyclopia, shortening in the anteroposterior axis, and developmental delay, associated with oxidative damage [25].

In the last three decades, HABs increased around the globe in distribution, frequency, and duration, with negative sanitary and economic impacts [26,27,28,29,30,31]. The main reasons are correlated with natural algal species dispersion by currents and winds and by direct anthropogenic activities, including the large eutrophication of coastal areas and leakage of species through ship ballast waters [32]. Also, climate changes play a key role in the expansion of algal species due to ocean acidification, alterations of temperature, and stratification entry of nutrients induced by precipitation and light [33].

In this context, blooms of dinoflagellates producing DSP toxins could negatively impact the marine ecosystem, as they could alter the dynamics and composition of planktonic communities, reduce biodiversity, and unbalance food webs. Thus, to assess the potential ecotoxicological impact of DSP toxins, OA, DTX-1, and DTX-2 were evaluated for their toxic effects towards *Artemia franciscana* as a model organism of marine zooplankton at the basis of the marine food web, considering that *Artemia* species are commonly used for aquatic ecotoxicological studies [34] and for bioaccumulation studies along the food web [35]. In particular, this study was aimed at investigating the adverse effects of these toxins towards *A. franciscana* by means of impaired cyst hatching, mortality of nauplii Instar I and adults, and biochemical responses related to oxidative stress and the antioxidant defense system, i.e., reactive oxygen species (ROS) production and activity of selected endogenous antioxidant enzymes (glutathione S-transferase, catalase, and superoxide dismutase).

The use of *Artemia* microcrustaceans in these studies is particularly advantageous due to many factors, such as their high adaptability to environmental conditions, short life cycle, high fecundity, bisexual/parthenogenetic reproduction strategies, small body size, wide geographic distribution, and adaptability to varied nutrient resources as they are non-selective filter feeders. Moreover, there is good knowledge on their ecology and physiology and *Artemia*-based tests guarantee reliability, rapidity, simplicity, feasibility, and convenience in terms of the low cost, reduction in test volumes, amount of produced waste, and space needed [36]. In particular, *Artemia franciscana* is largely used in ecotoxicology studies as a model of marine zooplankton species [34,36,37,38], both in short- and long-term toxicity tests [39].

## 2. Results

### 2.1. Artemia franciscana Cyst Hatching and Mortality of Nauplii or Adults After Exposure to DSP Toxins

**Figure 1:**
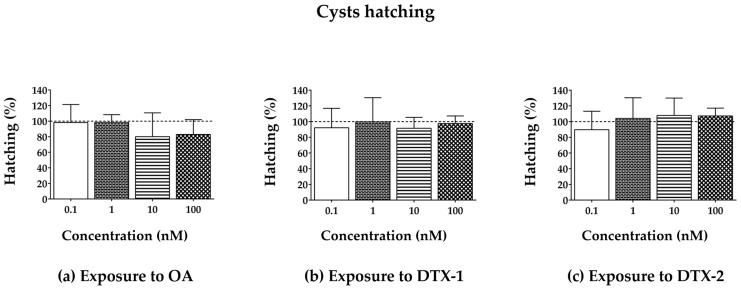
*A. franciscana* cyst hatching after the 96 h exposure to OA (**a**), DTX-1 (**b**), or DTX-2 (**c**) evaluated by a stereomicroscope. Cyst hatching data are presented as % of free-hatched nauplii with respect to the total number of cysts exposed to each toxin (the dashed line represents the hatching of cysts not exposed to the toxin; CTRL). Data are expressed as the mean ± SE of three independent experiments. Statistical differences: one-way ANOVA and Bonferroni post-test.

**Figure 2:**
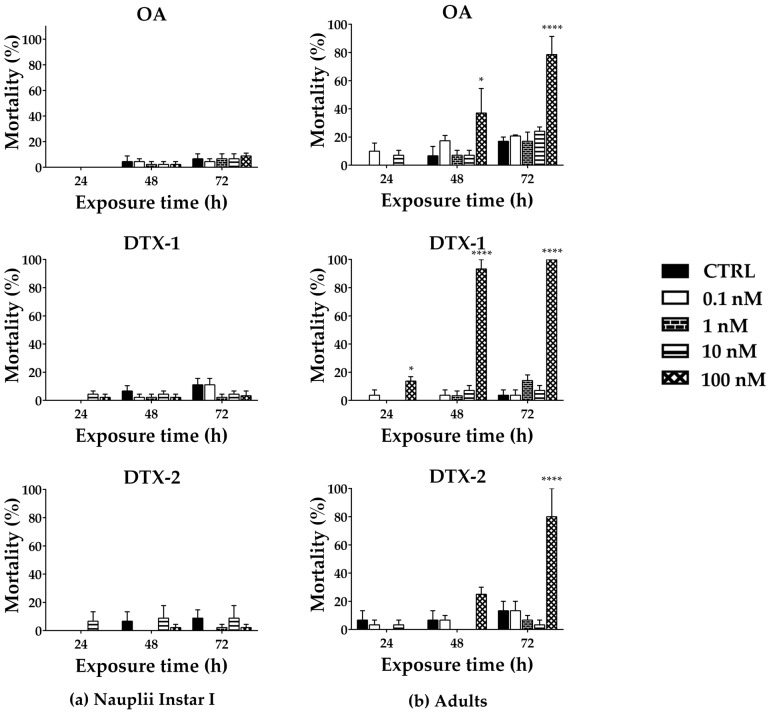
Mortality of *A. franciscana* nauplii Instar I (**a**) or adults (**b**) after 24, 48, and 72 h exposures to OA, DTX-1, or DTX-2. Data are presented as % of dead organisms with respect to the total number of exposed organisms and are expressed as the mean ± SE of three independent experiments. Statistical differences: *, *p* < 0.05; ****, *p* < 0.0001 (two-way ANOVA and Bonferroni post-test).

The toxic effects of OA, DTX-1, and -2 on *A. franciscana* were evaluated by means of cyst hatching after the 96 h exposure (0.1–100 nM; Figure 1) and their lethal effect towards nauplii Instar I or adults after 24, 48, and 72 h exposures (Figure 2). As a negative control (CTRL), *A. franciscana* cysts, nauplii Instar I, or adults were exposed to 0.1% MeOH.

*Artemia* cysts produce nauplii within 36 h after hydration, and exposure of *A. franciscana* cysts to OA, DTX-1, or DTX-2 for 96 h did not significantly reduce hatching, as compared to the negative control (Figure 1). Focusing on the toxins’ effect towards nauplii (Instar I) viability, no significant increase in mortality occurred after larvae exposure to each toxin for 24–72 h (Figure 2a). Conversely, exposure of adults to OA, DTX-1, or DTX-2 for 24-–72 h resulted in an increased mortality rate (Figure 2b). In particular, only the highest OA concentration (100 nM) significantly increased adults’ mortality after 48 and 72 h exposures (37%, *p* < 0.05, and 78%, *p* < 0.0001, respectively), as compared to the negative control (Figure 2b). Also, DTX-1 significantly increased adults’ mortality at the highest concentration (100 nM), with a significant effect even after the 24 h exposure: as compared to the negative control, it caused 14% (*p* < 0.05), 93% (*p* < 0.0001), and 100% (*p* < 0.0001) mortality increases after 24, 48, and 72 h, respectively (Figure 2b). Furthermore, only the 72 h exposure to the highest DTX-2 concentration (100 nM) significantly increased adults’ mortality (80%, *p* < 0.0001) (Figure 2b). These data indicate the following rank of lethal potency towards *A. franciscana* adults: DTX-1 > OA > DTX-2.

On the whole, these data indicate that adults are the most sensitive developmental stage of *A. franciscana* to OA, DTX-1, and DTX-2. Thus, in the subsequent study, adults were chosen to assess ROS production and antioxidant enzyme activity after exposure to each toxin (1–100 nM).

### 2.2. ROS Production and Antioxidant Enzyme Activity After Exposure to OA 

**Figure 3:**
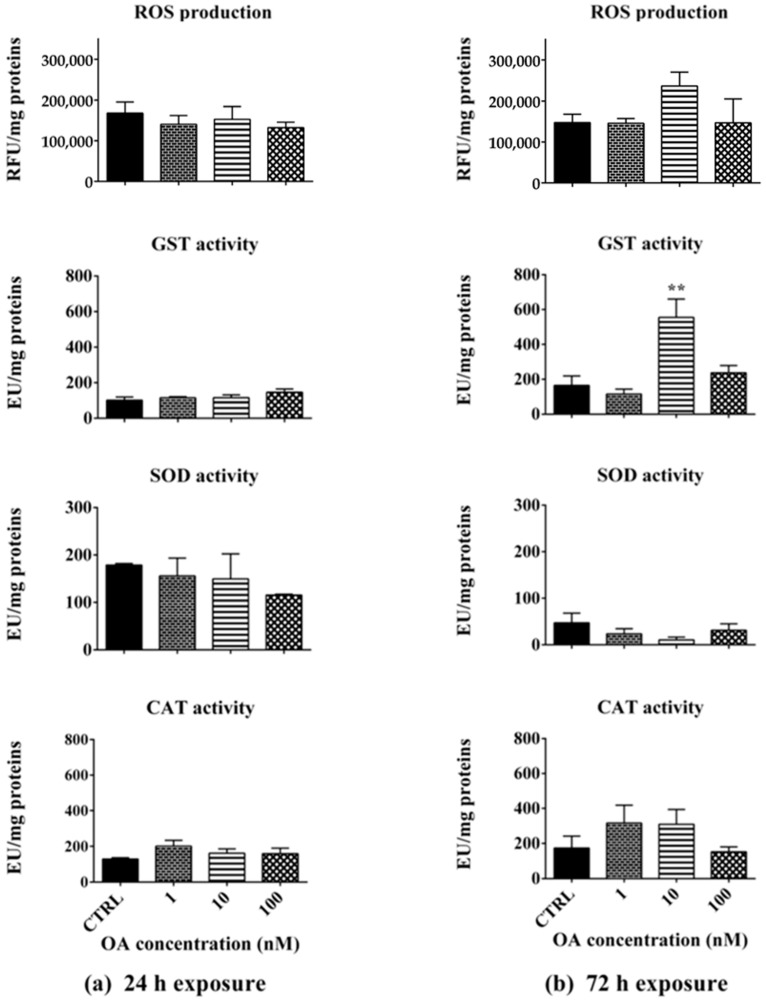
ROS production and activity of glutathione S-transferase (GST), superoxide dismutase (SOD), or catalase (CAT) in *A. franciscana* adults exposed to OA for 24 h (**a**) or 72 h (**b**). CTRL: negative control. ROS production: data are presented as Relative Fluorescent Units (RFUs) normalized on protein content (mg). Enzyme activity: data are presented as Enzymatic Units (EUs) normalized on protein content (mg). Data are expressed as mean ± SE of three independent experiments. Statistical differences: **, *p* < 0.01 (one-way ANOVA and Bonferroni post-test).

*A. franciscana* adults were exposed to OA for 24 or 72 h to assess the toxin ability to induce ROS production or impair endogenous antioxidant enzyme activity as biomarkers of oxidative stress. After the 24 h exposure, OA did not significantly influence ROS production or antioxidant enzyme activity, as compared to the negative control (Figure 3a). Prolonging the exposure to 72 h, only the concentration of 10 nM induced a non-significant increase in ROS production and a significant GST activity increase (239%; *p* < 0.01), SOD and CAT activities being unaffected (Figure 3b).

### 2.3. ROS Production and Antioxidant Enzyme Activity After Exposure to DTX-1

**Figure 4:**
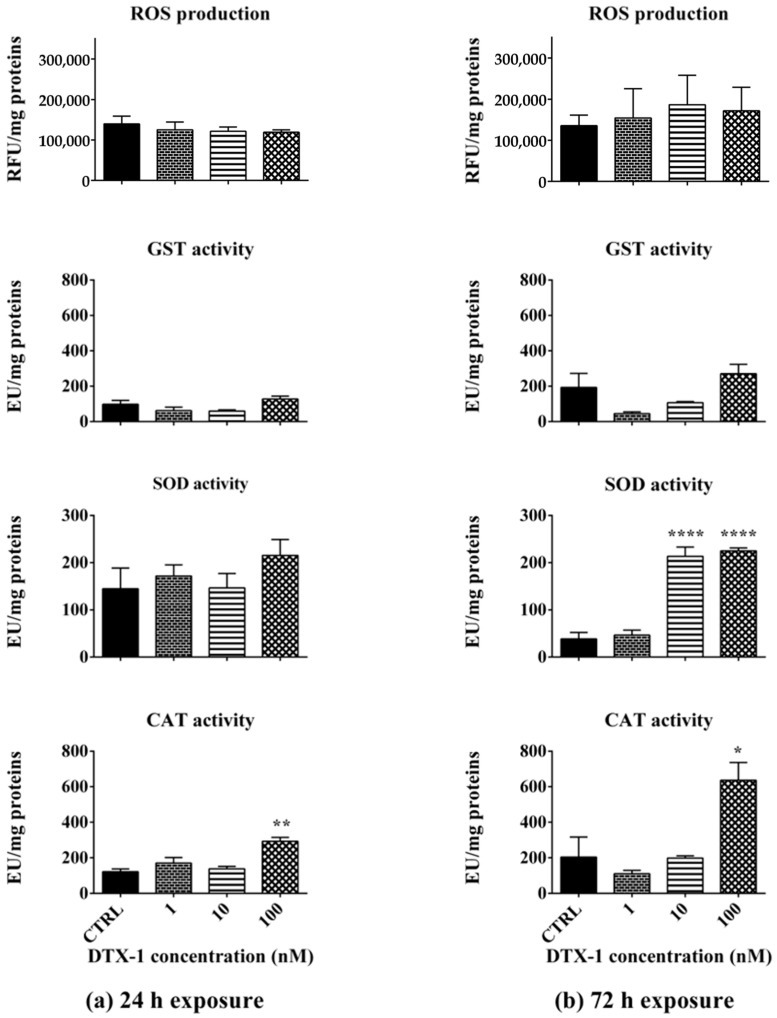
ROS production and activity of glutathione S-transferase (GST), superoxide dismutase (SOD), or catalase (CAT) in *A. franciscana* adults exposed to DTX-1 for 24 h (**a**) or 72 h (**b**). CTRL: negative control. ROS production: data are presented as Relative Fluorescent Units (RFUs) normalized on protein content (mg). Enzyme activity: data are presented as Enzymatic Units (EUs) normalized on protein content (mg). Data are expressed as mean ± SE of three independent experiments. Statistical differences: *, *p* < 0.05; **, *p* < 0.01; ****, *p* < 0.0001 (one-way ANOVA and Bonferroni post-test).

Adults’ exposure to DTX-1 for 24 h did not influence ROS production or GST and SOD activity. On the other hand, only its highest concentration (100 nM) significantly increased CAT activity (142%; *p* < 0.01), as compared to the negative control (Figure 4a). After the 72 h exposure, 10 nM and 100 nM DTX-1 increased SOD activity by 457% (*p* < 0.0001) and 488% (*p* < 0.0001), respectively. Moreover, its highest concentration (100 nM) also increased CAT activity by 211% (*p* < 0.05). ROS production and GST activity were not affected by the 72 h exposure to DTX-1 (Figure 4b).

### 2.4. ROS Production and Antioxidant Enzyme Activity After Exposure to DTX-2

**Figure 5:**
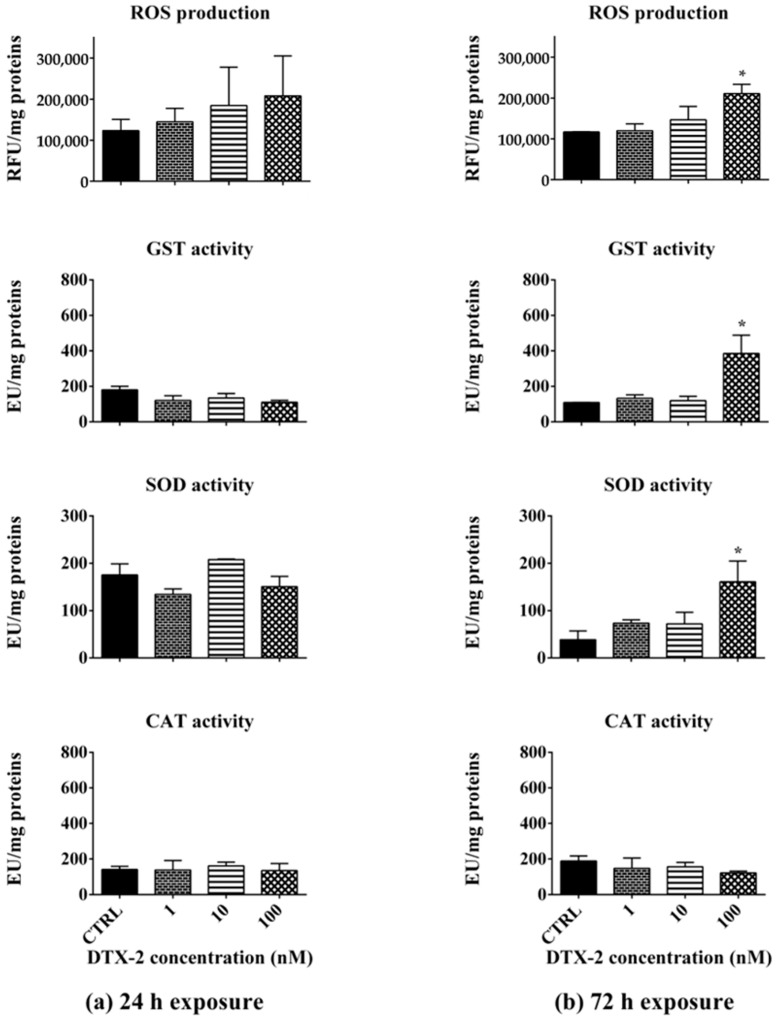
ROS production and activity of glutathione S-transferase (GST), superoxide dismutase (SOD), or catalase (CAT), in *A. franciscana* adults exposed to DTX-2 for 24 h (**a**) or 72 h (**b**). CTRL: negative control. ROS production: data are presented as Relative Fluorescent Units (RFUs) normalized on protein content (mg). Enzyme activity: data are presented as Enzymatic Units (EUs) normalized on protein content (mg). Data are expressed as mean ± SE of three independent experiments. Statistical differences: *, *p* < 0.05 (one-way ANOVA and Bonferroni post-test).

Adults’ exposure to DTX-2 for 24 h induced a not significant concentration-related increase in ROS production and did not affect the endogenous antioxidant enzymes’ activity (Figure 5a). After the 72 h exposure, the highest toxin concentration (100 nM) significantly increased ROS production (80%; *p* < 0.05) as well as the activity of GST (254%; *p* < 0.05) and SOD (320%; *p* < 0.05), without affecting CAT activity (Figure 5b).

## 3. Discussion

This study compared the toxic effects of OA and DTXs on the microcrustacean *Artemia franciscana*, considering *Artemia* species as representative organisms of marine zooplankton and their wide use as models for ecotoxicological studies [40,41,42,43], in particular for the marine environment [34] and for bioaccumulation studies along the food web [35]. This organism is suitable for acute or chronic toxicity studies, from hatching to nauplii at different developmental stages and adults, also taking advantage of the good knowledge of its biology and ecology. Moreover, it allows the possibility to analyze different physiological parameters, biomarkers of oxidative stress, and endpoints of toxicity, movement, and reproductive toxicity [39].

For these reasons, three DSP toxins (OA, DTX-1, and DTX-2) were investigated for their effects on *A. franciscana* by means of cyst hatching (96 h exposure) and a lethal effect towards nauplii stage I or adults (24–72 h exposure). The hatching assay revealed that none of the toxins significantly affected the percentage of nauplii produced by cysts. Similarly, the toxins did not significantly increase mortality of nauplii Instar I. On the contrary, each toxin significantly increased adults’ mortality with a different potency. In particular, exposure of adults to OA (100 nM) for 48 and 72 h significantly increased mortality (37% and 78%, respectively), but no significant effect was recorded after the 24 h exposure. Previously, Gong et al. [44] evaluated OA lethality towards *Artemia salina*, recording a reduced survival of both stage I nauplii and adults even after a 24 h exposure: the LC_50_ was 170.0 µg/L (C.I. 95% = 143.4–201.7 µg/L) for nauplii stage I and 186.4 µg/L (C.I. 95% = 156.1– 222.6 µg/L) for adults, which correspond to approximately 210 nM and 230 nM OA, respectively. However, the 24 h exposure of *A. salina* to the low OA concentrations (approximately 30 nM, 62 nM, and 124 nM, within the range of 0.1–100 nM used in our study or slightly higher) exerted a low toxic effect (<20% mortality), comparable to that recorded in our study. DTX-1 was also lethal for *A. franciscana* adults only at the highest concentration (100 nM), but it significantly increased mortality even after the 24 h exposure (14% mortality), reaching 93% and 100% mortality after 48 and 72 h exposures, respectively. A significant increase in adults’ mortality (80%) was also induced by the 72 h exposure to the highest DTX-2 concentration (100 nM). Among the tested DTXs, only DTX-1 was recently assessed for its toxicity towards *Artemia:* after a 48 h exposure, Zhang et al. recorded an increased mortality of *A. salina* nauplii (stage not specified), with an LD_50_ of 0.0819 µg/mL (about 100 nM) [45], in contrast with our findings showing no effect on nauplii viability. This discrepancy could be due to interspecies variation in sensitivity to the toxin and/or to different developmental stages of nauplii (not specified by Zhang et al.) used in the two studies [36,39].

Considering mortality after the 72 h exposure as an endpoint of toxicity, the present study shows that *A. franciscana* adults are the most sensitive developmental stage to OA, DTX-1, and DTX-2. Indeed, considering the mortality rate of adults after exposure to the lethal toxins’ concentration (100 nM) for 24, 48, and 72 h, DTX-1 appeared to be the most toxic analog, with the most rapid lethal effect (14–100% mortality after 24–72 h exposure), followed by OA (37–78% mortality after 48–72 h exposure) and DTX-2 (80% mortality only after 72 h exposure). The low sensitivity of nauplii Instar I to these toxins could be related to the incomplete morphological and functional development of the digestive tract at this stage [46] that could impair the gastrointestinal absorption of these compounds and, consequently, limit their toxic effects. Our observation agrees with those of previous studies on *Artemia* species, showing that stage I nauplii are less sensitive than adults to the dinoflagellate toxin palytoxin [47] or to other xenobiotics [48,49,50].

Considering these results, biochemical markers related to oxidative stress and the antioxidant defense system were also evaluated as endpoints of toxicity in adults exposed to each toxin for 24 and 72 h. The results showed that OA or DTX-1 did not increase ROS production in adults after 24 h nor after the 72 h exposure, but they influenced the activities of some endogenous defense antioxidant enzymes. Specifically, the 72 h exposure to OA significantly increased GST activity, suggesting an induction of the antioxidant defense system able to counteract ROS accumulation. On the other hand, adult exposure to DTX-1 for 24 and 72 h increased CAT activity, specific for hydrogen peroxide metabolism [51]. In addition, the 72 h exposure to DTX-1 significantly increased the activity of SOD, an enzyme involved in the conversion of superoxide anion radicals to hydrogen peroxide and oxygen [52]. Thus, both SOD and CAT activation may imply an efficient neutralization system against superoxide anion radicals and hydrogen peroxide, able to limit ROS accumulation, at least after a 72 h exposure to DTX-1. The toxic effect of OA and its ability to induce oxidative stress and increase endogenous antioxidants were also previously recorded in aquatic vertebrates, such as seabream (*Sparus aurata*), in which a 4 or 24 h exposure to the toxin (7.5 μg/mL, corresponding to about 9300 nM) increased CAT activity and the total glutathione level [22]. Furthermore, a significant increase in ROS production was recorded in medaka larvae (*Oryzias melastigma*, at 12 days) exposed to OA for 48 h (0.38 μg/mL, corresponding to about 470 nM), as well as in CAT activity in 1-month-old larvae exposed for 96 h to OA (0.095 and 0.38 μg/mL, equal to about 120 nM and 470 nM) [24]. On the contrary, up to a 96 h exposure of Japanese medaka larvae (*Oryzias latipes*, at 121 h post fertilization) to higher concentrations of OA or DTX-1 (1–30 μg/mL, equal to about 1240–37,260 nM) resulted in a decreased activity of endogenous antioxidant enzymes (CAT, SOD, glutathione peroxidase, and glutathione reductase) and increased oxidative damage [53].

Among the tested DSP toxins, only DTX-2 significantly increased ROS production: the 72 h exposure to the highest concentration (100 nM) increased ROS production by 80%, a phenomenon associated with a high mortality of *A. franciscana* adults. Furthermore, the 72 h exposure to the same concentration significantly increased GST and SOD activities, suggesting an activation of the antioxidant defense system, albeit in an insufficient way to neutralize the high level of produced ROS.

On the whole, our data indicate that the order of lethal potency of the three DSP toxins towards *A. franciscana* is DTX-1 > OA > DTX-2, in agreement with previous in vitro findings on cytotoxicity towards mammalian cells [54,55]. Comparing the effects of the three toxins, their lethal potency appears to not be directly related to their ability to increase ROS production or to activate endogenous antioxidant enzymes controlling the level of oxidative stress. Thus, also, other mechanisms related to protein phosphatase inhibition by these toxins might be involved in toxic effects towards *A. franciscana*. In this regard, according to Huhn et al. [56], the different toxic potency of the three toxins could be related to differences in the affinity for their main molecular target, i.e., protein phosphatase 2A (PP2A), a fundamental enzyme for many cellular processes, including cellular division and differentiation or neuronal activity. In fact, DTX-1 and DTX-2 possess a 35-methyl group in equatorial and axial positions, respectively, whereas OA is not methylated in this position. While the equatorial 35-methyl of DTX-1 appears to favor the toxin interaction with the PP2A binding site, the axial 35-methyl of DTX-2 does not favor this interaction with PP2A, which leads to a decreased affinity for the enzyme.

In conclusion, the results of this study indicate a potential ecotoxicological impact of OA, DTX-1, and DTX-2, associated with a possible reduction in *Artemia franciscana* populations and additional adverse impact on other *Artemia* species. Given that these microcrustaceans are representative organisms of the marine zooplankton, which plays an important role in the marine food web, reduction in their populations could have negative impacts on organisms at higher trophic levels. However, it has to be underlined that the toxic effects of OA, DTX-1, and DTX-2 towards *A. franciscana* were recorded at concentrations higher than that to which these organisms would typically be exposed in the marine environment. In fact, OA concentrations detected in seawater ranged from 1 nM (Spain) to 0.0019 nM (China), whereas a concentration of 0.00038 nM was detected in marine phytoplankton (China) [57,58]. Furthermore, DTX-1 concentrations in seawater were previously detected at levels ranging from 0.01 nM to 0.0004 nM (China) [57,59], and those of DTX-2 ranged from 0.0003 nM [60] to 0.0019 nM (China) [61].

## 4. Conclusions

In the present study, the potential ecotoxicological impact of three marine algal toxins frequently detected in recent decades during HABs (OA, DTX-1, and DTX-2) was assessed using *Artemia franciscana* as a model organism. While no significant effect on *A. franciscana* cyst hatching and nauplii Instar I viability was recorded, the 72 h exposure to each toxin significantly increased mortality in adults, DTX-1 being the most potent, followed by OA and DTX-2. Hypothesizing oxidative stress as a possible critical event in the adverse effects, the evaluation of ROS production and endogenous antioxidant enzyme activity showed that only DTX-2 increased ROS levels after the 72 h exposure, whereas each toxin influenced the antioxidant enzyme activity with a different profile of action. These findings indicate a not direct relation between the toxin lethality and their ability to induce oxidative stress, suggesting that other mechanisms might be involved in the toxicity towards *A. franciscana*, such as those related to protein phosphatase inhibition. Finally, although the toxic effects occurred at concentrations higher than those detected in marine environments, the increasing frequency and the geographic distribution of HABs could lead to higher environmental toxin concentrations. Such events could have ecotoxicological impacts related to a reduced population of *Artemia* species that in turn could negatively impact other organisms at a higher trophic level.

## 5. Materials and Methods

### 5.1. Toxins

OA and DTX-1 were extracted from cultures of *Prorocentrum lima* strain 1036 while DTX-2 was extracted from contaminated shellfish (*Mytilus edulis*) collected in 2010 from the Southwest of Ireland, as previously reported [62]. The purity of each toxin (>95%) was confirmed by LC-MS/MS and NMR, as previously described [62].

### 5.2. Hatching and Breeding of Artemia franciscana

Dehydrated cysts of *A. franciscana* as well as the products for the hatching and breeding of this crustacean were purchased from Hobby (Gelsdorf, Germany), unless otherwise specified. The taxonomic identification of *A. franciscana* was previously confirmed by PCR sequencing [47,63]. To obtain Instar I stage larvae, approximately 300 mg cysts were hatched in a specific *Artemia* hatchery dish, using 750 mL artificial seawater (for each liter of deionized water, 36 g of Optimum Sea basic salt; Wave, Italy) in the constant presence of artificial light, at 25 °C for 24 h. Subsequently, larvae were separated from unhatched cysts, transferred into fresh artificial seawater, and (i) used for the mortality test or (ii) transferred into a beaker and bred in artificial seawater for 21 days to obtain *A. franciscana* adults. During the first 10 days after hatching, *A. franciscana* organisms were fed three times a week with liquid (Liquizell; Hobby; Gelsdorf, Germany), and subsequently solid food (Mikrozell; Hobby; Gelsdorf, Germany), specific for *Artemia* culturing. The organisms were maintained at 25 °C under a 16:8 h light/dark cycle.

### 5.3. Toxicity Assays

The toxin solutions, in 100% MeOH, were diluted in artificial seawater containing 0.1% MeOH as the final concentration. The toxin concentrations used for the toxicity assays ranged from 0.1 nM to 100 nM, with a dilution factor of 10, maintaining as constant the final concentration of MeOH (0.1%). For each test, the negative control (CTRL) was obtained exposing the organisms to artificial seawater containing 0.1% MeOH, a solvent concentration that did not affect viability of *A. franciscana* up to the longest time of exposure considered (72 h), as shown from preliminary experiments.

#### 5.3.1. Hatching Assay

Hatching tests were performed according to Migliore et al. [64]. Briefly, 10 cysts were transferred into each well of a 96-well plate containing a final volume of 200 µL artificial seawater and exposed with the toxins, or 0.1% MeOH (CTRL), up to 96 h. The amount of nauplii was counted every 24 h up to 96 h under the stereomicroscope (3× of magnification; Kyowa; Tokyo, Japan) and hatching was calculated by the ratio between the number of free hatched nauplii and the number of exposed cysts. Results are represented as % with respect to the negative control after the 96 h exposure.

#### 5.3.2. Mortality Assay on Nauplii Instar I

After cysts hatched in the hatchery dish, 5 nauplii Instar I were transferred to each well of a 96-well plate containing a final volume of 200 μL artificial seawater, and exposed to the toxins or 0.1% MeOH (CTRL) for 24, 48, and 72 h. During the mortality test, nauplii were not fed. According to Zulkifli et al. [65], animals that did not present any movement for 10 s, observed under the stereomicroscope (Kyowa; Tokyo, Japan, at 3× magnification) were considered dead. The amount of dead nauplii was counted after 24, 48, and 72 h exposures. Mortality, expressed as %, was calculated by the ratio between dead nauplii and the total number of nauplii in each sample.

#### 5.3.3. Mortality Assay on Adults

After 21 days of breeding, *A. franciscana* adults (10) were transferred to each well of a 24-well plate and exposed to the toxins or 0.1% MeOH (CTRL) in a final volume of 1.5 mL artificial seawater for 24, 48, and 72 h. Adults were fed with solid food 24 h before the test and 24 h after. Adults were considered dead in the case of the absence of movement for 10 s, as observed under the stereomicroscope (Kyowa; Tokyo, Japan, at 1× magnification). Mortality was calculated as the % ratio between dead organisms and the total number of organisms in each sample.

### 5.4. Sample Preparation for ROS Production and Antioxidant Enzyme Activity Assays

To assess the potential induction of oxidative stress by OA and DTXs, ROS production and endogenous antioxidant enzyme activity were evaluated in adults exposed to each toxin (1–100 nM) for 24 and 72 h. *A. franciscana* samples were prepared as previously reported [47,63]. Briefly, 50 adults were transferred into each well of a 6-well plate, exposed with the toxins or 0.1% MeOH (CTRL) in a final volume of 4.5 mL. After 24 or 72 h exposures, the organisms were washed 3 times in 50 mM phosphate buffer, pH 7 (composed of sodium phosphate dibasic and sodium phosphate monohydrate; Sigma-Aldrich; Milan, Italy) added with 5.0 mM ethylenediaminetetraacetic acid (Sigma-Aldrich; Milan, Italy). The organisms of each group were transferred into an Eppendorf tube containing a final volume of 400 μL of the same buffer and homogenized for 15 s using an immersion sonicator (Ultrasonic processor UP50H; Hielscher; Teltow, Germany). After centrifugation at 15,000× *g* at 4 °C for 25 min, the supernatant was collected and preserved at −80 °C.

### 5.5. Reactive Oxygen Species Production Assay

ROS production in *A. franciscana* adults exposed to each toxin or 0.1% MeOH (CTRL) for 24 h and 72 h was measured using the fluorogenic probe 2’,7’-dichlorofluorescin diacetate (DCFDA; Sigma-Aldrich; Milan, Italy), as previously reported [47,63]. Briefly, in each well of a 96-well plate, 175 μL phosphate-buffered saline (PBS) was mixed with 5 μL sample supernatant and 20 μL 4.0 × 10^−7^ M DCFDA solution. Two blanks were included: the first was created by mixing 175 μL PBS with 5 μL 50.0 mM phosphate buffer, pH 7 (instead of the sample), and 20 μL 4.0 × 10^−7^ M DCFDA; the second blank consisted of 195 μL PBS and 5 μL sample supernatant (none of these blanks produced a significant increase in fluorescence). The plate was incubated at 37 °C in the dark for 30 min, and the fluorescence was read at 485 nm and 520 nm (excitation and emission wavelengths, respectively) using a microplate reader (FLUOstar Omega; BMG LABTECH; Offenburg, Germany). All the samples were tested in triplicate. Results were reported as mean Relative Fluorescence Units (RFUs) measured at the microplate reader and normalized on protein content for each sample.

### 5.6. Antioxidant Enzyme Activity Assays

Glutathione S-Transferase (GST), superoxide dismutase (SOD), and catalase (CAT) activity in *A. franciscana* adults exposed to each toxin or 0.1% MeOH (CTRL) for 24 and 72 h was measured using supernatant samples prepared as reported above.

#### 5.6.1. Glutathione S-Transferase Activity Assay

The GST activity assay was based on the methods described by Habig et al. [66], as previously reported [67], adapted to *Artemia* adults. Briefly, the reaction mixture was prepared using 1.3 mM reduced L-glutathione (Sigma-Aldrich; Milan, Italy) and 1.3 mM 1-chloro-2,4-dinitrobenzene (CDNB; Sigma-Aldrich; Milan, Italy) in 100 mM phosphate buffer, pH 6.5. In each well of a 96-well plate, 75 μL of the reaction mixture was added to 25 μL sample supernatant or blank and the absorbance was read immediately at 304 nm, and every 30 s for 3 min, using a microplate reader (FLUOstar Omega; BMG LABTECH; Offenburg, Germany). The blank was prepared in the same way, without the probe (CDNB), being replaced with the phosphate buffer.

#### 5.6.2. Superoxide Dismutase Activity Assay

According to Zhao et al. [68], the SOD activity was quantified by measuring the enzyme ability to inhibit the photochemical reduction of nitro blue tetrazolium (NBT). The assay was performed using 96-well plates, adding to each well 25 μL of the sample supernatant or blank and 75 μL of the reaction mixture. The reaction mixture was composed of 50 mM phosphate buffer, pH 7.8 (composed of Na_2_HPO_4_ and NaH_2_PO_4_H_2_O; Sigma-Aldrich; Milan, Italy), containing 140.0 mM L-methionine (Sigma-Aldrich; Milan, Italy), 0.7 mM NBT (Sigma-Aldrich; Milan, Italy), 0.02 mM Riboflavin (Sigma-Aldrich; Milan, Italy), and 0.1 mM Na_2_EDTA (Sigma-Aldrich; Milan, Italy). The blank was produced by replacing 25 μL of the sample supernatant with the same volume of the phosphate buffer. The 96-well plate was incubated at room temperature for 30 min under a 900-lumen lamp to allow the photochemical reaction; then, the absorbance was read at 560 nm using a microplate reader (FLUOstar Omega; BMG Labtech; Ortenberg, Germany).

#### 5.6.3. Catalase Activity Assay

CAT activity in *A. franciscana* adults exposed to each toxin or 0.1% MeOH (CTRL) for 24 and 72 h was quantified as previously described by Zhao et al. [68] on earthworms, adapting the assay for *Artemia*. Briefly, in each well of a 96-well UV-transparent plate, 4 μL of the sample supernatant and 46 μL 50 mM phosphate buffer, pH 7.0, were added in duplicate. Afterwards, 50 μL of a 20.0 mM H_2_O_2_ solution (Sigma-Aldrich; Milan, Italy) was added to obtain a final H_2_O_2_ concentration in the well of 10 mM. For each sample, the blank was produced by replacing 50 μL of the hydrogen peroxide solution with an equal volume of the phosphate buffer. Then, the plate was read at a wavelength of 240 nm every 30 s for 3 min, using a microplate reader (FLUOstar Omega; BMG Labtech; Ortenberg, Germany).

#### 5.6.4. Antioxidant Enzymes’ Activity Calculation

The enzyme (GST, CAT) activities were expressed as Enzyme Units (EUs), calculated by the following formula:

$$EU=\frac{\frac{\Delta Absorbance\;}{min}}{\varepsilon *l}*\frac{V\;total}{V\;supernatant}$$

where ε was 9 600 M^−1^ × cm^−1^ for GST and 43.6 M^−1^ × cm^−1^ for CAT. The enzyme activity was normalized on protein content (EU/mg proteins) of each sample and the relevant measure units correspond to nmol/min for GST and μmol/min for CAT.

One EU of SOD was defined as the enzyme amount needed to inhibit the photochemical reduction of NBT by 50%, and SOD activity was reported as EU/mg proteins.

Protein content in each *A. franciscana* sample prepared to determine ROS production and antioxidant enzyme activity was quantified using a NanoDrop 2000 (ThermoFisher Scientific; Milan, Italy) at 280 nm.

### 5.7. Statistical Analysis

All the results are expressed as the mean ± standard errors of the mean (SEs) of at least three independent experiments.

Depending on the biological assays, data were analyzed by a one- or two-way analysis of variance (ANOVA) and Bonferroni post-test, using GraphPad Prism software version 6. Significant differences were considered for *p* < 0.05.

## Data Availability

The original contributions presented in this study are included in the article. Further inquiries can be directed to the corresponding author(s).
